# Characterization and Benchmark of a Novel Capacitive and Fluidic Inclination Sensor

**DOI:** 10.3390/s21238030

**Published:** 2021-12-01

**Authors:** Adrian Schwenck, Thomas Guenther, André Zimmermann

**Affiliations:** 1 Institute for Micro Integration (IFM), University of Stuttgart, Allmandring 9b, 70569 Stuttgart, Germany; Thomas.Guenther@ifm.uni-stuttgart.de (T.G.); Andre.Zimmermann@ifm.uni-stuttgart.de (A.Z.); 2 Hahn-Schickard, Allmandring 9b, 70569 Stuttgart, Germany

**Keywords:** inclination sensor, tilt sensor, capacitive sensor, fluidic sensor, PCB-based sensor, MID sensor, benchmark, Allan deviation, temperature stability, characteristic curve

## Abstract

In this paper, a fluidic capacitive inclination sensor is presented and compared to three types of silicon-based microelectromechanical system (MEMS) accelerometers. MEMS accelerometers are commonly used for tilt measurement. They can only be manufactured by large companies with clean-room technology due to the high requirements during assembly. In contrast, the fluidic sensor can be produced by small- and medium-sized enterprises (SMEs) as well, since only surface mount technologies (SMT) are required. Three different variants of the fluidic sensor were investigated. Two variants using stacked printed circuit boards (PCBs) and one variant with 3D-molded interconnect devices (MIDs) to form the sensor element are presented. Allan deviation, non-repeatability, hysteresis, and offset temperature stability were measured to compare the sensors. Within the fluidic sensors, the PCB variant with two sensor cavities performed best regarding all the measurement results except non-repeatability. Regarding bias stability, white noise, which was determined from the Allan deviation, and hysteresis, the fluidic sensors outperformed the MEMS-based sensors. The accelerometer Analog Devices ADXL355 offers slightly better results regarding offset temperature stability and non-repeatability. The MEMS sensors Bosch BMA280 and TDK InvenSense MPU6500 do not match the performance of fluidic sensors in any category. Their advantages are the favorable price and the smaller package. From the investigations, it can be concluded that the fluidic sensor is competitive in the targeted price range, especially for applications with extended requirements regarding bias stability, noise, and hysteresis.

## 1. Introduction

**Figure 1:**
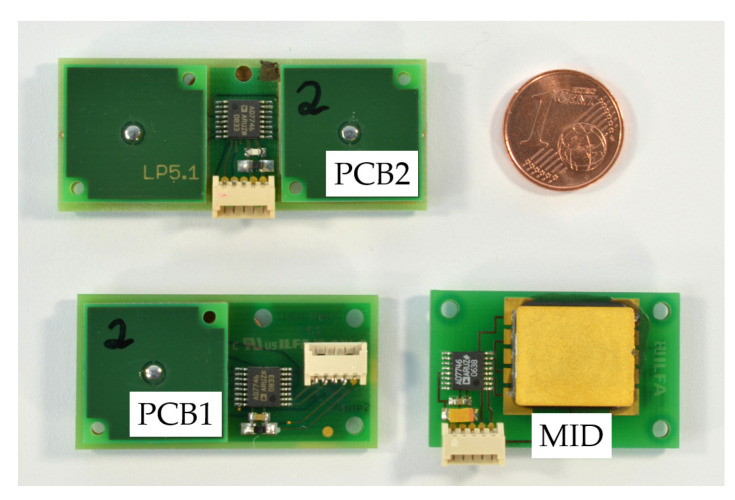
Three variants of the fluidic capacitive inclination sensors investigated in this work with different manufacturing processes. The different variants are described in detail in Section 3.1.1. MID Sensor and Section 3.1.2. PCB Sensor.

In this paper, a fluidic inclination sensor with capacitive readout is investigated (Figure 1). To form the cavity for a dielectric fluid, two different packaging technologies are used. These are molded interconnect devices (MIDs) and stacked printed circuit boards (PCBs).

Inclination sensors measure their relative position to the earth’s gravity field and are used in a wide field of applications, e.g., consumer electronics, inertial navigation systems, surveying technology, construction industry, and other applications that need a gravity reference [1,2,3]. Inertial navigation systems are used in ground-, air-, and sea-based vehicles and robots. In consumer electronics, popular use cases are display orientation sensing in smartphones and position detection in handheld controllers for video games.

Capacitive, optical, resistive, thermal, and inductive operating principles are typically used for inclination sensors. An overview is given in [4]. They can be precision-engineered or micromechanical systems (MEMS). Only MEMS accelerometers are available in the targeted price and size range [2,3]. MEMS accelerometers can be used as inclinometers by measuring the projection of the earth’s acceleration on the sensitive axis [2]. They are, with a turnover of USD 1134 million in 2020, among the MEMS devices with the highest market volume [5]. MEMS accelerometers can be classified into spring-mass, resonant element, and thermal systems [6].

Resonant-element-based sensors are described in [7,8] and are produced by the companies Seiko Epson [9], iXBlue [10], and Honeywell [11] for example. They are spring-mass-based systems and use a quartz resonator to measure the tension in the spring. Any change in the tension causes a frequency shift of the resonator. Another possibility is to measure the amplitude change in localization-based resonant MEMS accelerometers instead of the frequency [12].

Thermal MEMS accelerometers use convective heat transfer in a hermetically sealed and gas-filled cavity between a heater and temperature sensors mounted symmetrically next to it. Acceleration causes asymmetry in the heat distribution, which can be measured by the temperature sensors [1]. They are described, for example, in [13] and commercially available from the company Memsic [14].

Resonant and thermal systems are not further regarded in this work, as they are expensive and less common, respectively.

For low-g measurement with MEMS accelerometers, the most common measurement principle is the capacitive detection of the position of a seismic mass in a spring-mass system [2]. For higher accelerations, e.g., for crash detection in airbag systems for cars, piezoresistive measurement is often used [1]. Piezoelectric accelerometers are not suitable for inclination measurement, as they are not able to measure static acceleration [1].

The fluidic measurement principle in inclination sensors is often combined with capacitive, resistive, or optical readout. Examples of fluidic capacitive sensors in research can be found in [15,16,17,18], and commercially available sensors are fabricated for example by SEIKA Mikrosystemtechnik GmbH [19]. Fluidic principles with resistive readout are used in sensors from Jewell Instruments [20], Spectron Glass & Electronics Inc. [21], and The Fredericks Company [22] for example. An optical principle to detect a fluidic surface is used in the sensor Nivel 230 from Leica Geosystems [23].

Silicon-based MEMS acceleration sensor systems are dominating the inclination sensor market. Manufacturing technologies are surface micromachining (SMM) for low-cost and bulk micromachining (BMM) for high-end MEMS.

**Figure 2:**
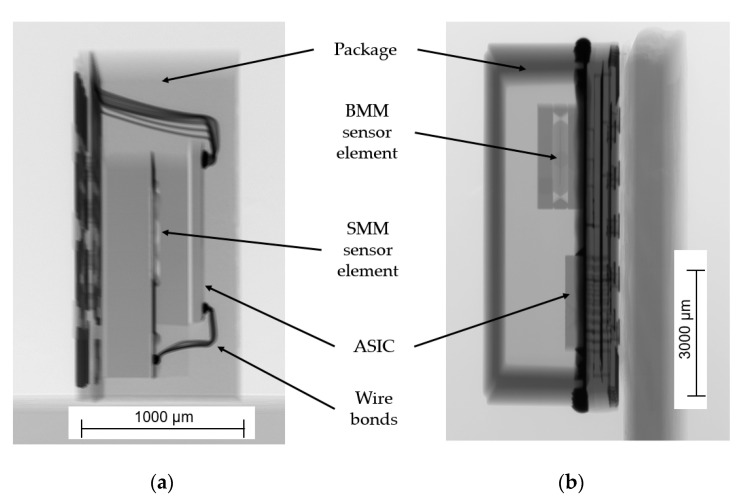
X-ray images of silicon MEMS acceleration sensors (not to scale). Images were made with a Nikon HMXST 160 computed tomography scan (CT): (**a**) Bosch BMA 280 with a three-axis SMM sensor element and a package size of 2 × 2 × 0.95 mm^3^; (**b**) Safran Colibrys SI1003.A with a one-axis BMM sensor element in an LCC20 package with a size of 9 × 9 × 3.3 mm^3^.

The SMM process is CMOS compatible and offers the possibility for hybrid integration of the sensor structure and the electronic circuits [24]. Another possibility is to stack silicon layers for the sensor structure and the electronic circuit over each other and provide the electrical connection with wire bonding (Figure 2a).

Silicon MEMS, fabricated with bulk micromachining (BMM), offer a better signal-to-noise ratio, as higher seismic masses are possible (Figure 2b). On the other hand, less common technologies such as silicon fusion bonding, wet anisotropic etching (KOH), or deep reactive ion etching (DRIE) are necessary for the production of BMM MEMS [25].

Most MEMS accelerometers are designed as open-loop systems; the deflection of a seismic mass is measured capacitively and used directly as the output signal. To increase the linear measurement range, closed-loop systems are used. In closed-loop accelerometers, a force, e.g., an electrostatic or magnetic force, is applied on the seismic mass to counteract the deflection of the seismic mass and to keep it in the zero position. In this case, the control loop signal is used as the output signal of the sensor. Thus, the sensor system complexity increases [25].

Another method to measure the deflection of the seismic mass is the quantum tunneling effect. These accelerometers can be fabricated in BMM or SMM and can be operated in open-loop or closed-loop configuration [26].

MIDs are injection molded, three-dimensional circuit carriers and most often made of thermoplastics [27]. Recent developments are using thermoset materials [28] or ceramics [29] as well. Metallic electronic circuits can be fabricated on the substrate in different ways. Examples are laser-based processes, aerosol-jet printing, hot embossing, and two-shot molding [30]. On MIDs, all conventional integrated circuit packaging technologies are possible. MIDs are used commercially for a wide range of applications. They can be used to align silicon sensors [31] or light-emitting diodes (LEDs) [32] freely in three dimensions, act as housing with integrated electrical connections [30], or form antenna structures [30]. In this work, the MID itself is used to form the sensing element.

PCBs do not offer three-dimensional structures as MIDs, but they can be used to form sensor elements as well [33]. Advantages are the wide and cheap availability and the low initial costs for production. Thus, it is possible to produce small series economically.

**Theorem** **1.** *Fluidic capacitive inclination sensors can compete in terms of price and performance with the commonly used BMM MEMS accelerometers in tilt measurement applications*.

## 2. Materials and Methods

### 2.1. Benchmark Sensors

As MEMS accelerometers are most commonly used for tilt measurement in the low-cost market, several commercially available sensors were selected as the benchmark. They are all open-loop systems. Typical examples in the lowest price segment are Bosch BMA280 and TDK InvenSense MPU6500. They are typically used in mobile phones for orientation detection. A significantly higher-performance and higher-priced sensor is ADXL355 from Analog Devices. Some important technical data are compared in Table 1. An additional sensor fabricated in BMM, Safran Colibrys SI1003, is added for comparison. BMM sensors are not further considered, as the costs of several hundred euros are much higher than the targeted costs of the fluidic capacitive inclination sensors.

**Figure 3:**
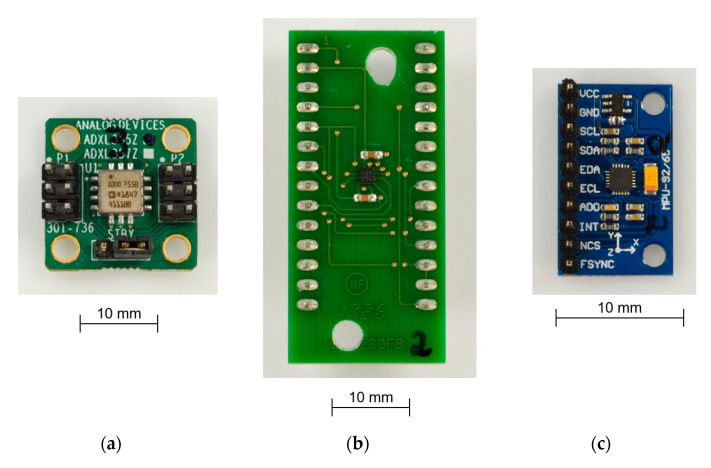
Sensors with evaluation boards used in this work (not to scale): (**a**) Analog Devices ADXL355Z; (**b**) Bosch BMA 280; (**c**) TDK InvenSense MPU6500.

SMM MEMS accelerometers are in a comparable price range; therefore, the three examples in Table 1 and Figure 3 are used as a reference in this paper.

The sensors, fluidic and MEMS, are compared at the same data rate of 5 Hz. As the MEMS sensors do not offer that data rate by default, 15 Hz was selected, and three values each were averaged.

### 2.2. Measurement Equipment

**Figure 4:**
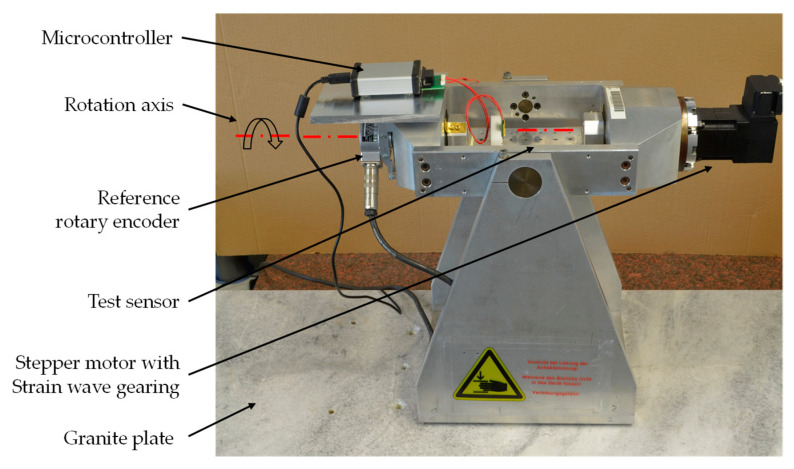
Test bench for measurement of characteristic curve.

A specially constructed test bench was used to measure the characteristic curve of the sensors (Figure 4). A rotary encoder Heidenhain ERN 480 with 5000 line counts was used as the reference signal. The rotary encoder offers a system accuracy of 1/20 of the grating period, which corresponds to 0.0036° [42]. To adjust the horizontal position, a tilt sensor Leica Geosystems Nivel230 with an accuracy of 0.00056° [23] was used. Both devices were calibrated by the respective manufacturer.

Power was supplied to the sensors with a Hameg HM 8142 power supply unit and a Linear Technologies LT3042 low noise low-dropout regulator (LDO).

**Figure 5:**
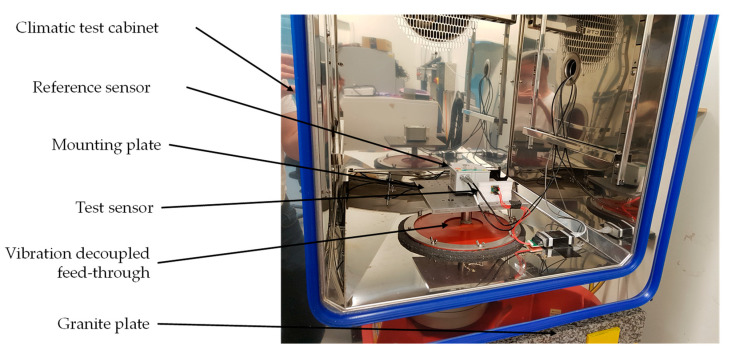
Climatic test chamber with mounting device for the sensors.

To conduct the measurement of the Allan deviation and the temperature stability, the sensors were placed in a climatic test chamber (Figure 5). In the climatic test chamber, the sensors were mounted on a decoupled reference plate. This ensures that the vibrations from the cooling of the climatic chamber do not affect the measurements, and it guarantees a stable horizontal alignment. The reference plate was connected to a granite plate, which is located under the climatic test chamber through an opening in the bottom of the climatic test chamber. Soft silicone mats were used for thermal isolation in the feedthrough. A reference sensor Leica Nivel 230 was mounted on the reference plate as well, and the signals were recorded during the measurements. The climatic test chamber was manufacturer calibrated.

### 2.3. Calibration

Before the measurements of the characteristic curves, all sensors were one-time calibrated with the test bench. The calibration includes several measuring points at room temperature. The calibration was used to calculate the measured angle values from the measured capacitance values for the fluidic sensors and from the measured acceleration values for the accelerometers. With a linear calibration for the fluidic sensor, and a two-point calibration for the accelerometers, simple methods were intentionally chosen, as the aim of the benchmark is to compare the raw sensor signals and not different calibration methods. The evaluation also compares characteristic values that are mostly independent of the calibration. A temperature calibration was not applied to any of the sensors. In the targeted market, a temperature calibration of individual sensors is unusual due to the high effort. If systematic temperature errors are present, a correction is possible, as no additional calibration effort is needed.

#### 2.3.1. Acceleration Sensors used as Inclination Sensors

A simple method to calibrate acceleration sensors for the application as inclination sensors is the one-point calibration [43]. It only calibrates the offset value, not the scale factor [43]. The two-point calibration [43] calibrates the gain as well. More sophisticated methods to calibrate three-axis acceleration sensors are three-point tumble sensor calibration [44], six-point tumble sensor calibration [45], and ellipsoid fitting [46].

As this work focuses on characteristic values of the sensors, which are independent of the calibration and only two of the three accelerometer axes are used, the two-point calibration was chosen.

To perform the two-point calibration the sensors were mounted on the test bench. The test bench was moved to the +90° position, and the measured acceleration value Accx(+90°) in g for the *X*-axis was read out. Afterwards, the measured value Accx(−90°) for the *X*-axis was read out at the −90° position. For the calibration of the *Y*-axis, the test bench was moved to the positions 0° and 180°, and the acceleration values Accy(0°) and Accy(180°) were read out. Offset and gain were calculated using Equations (1)–(4) [43].

(1) $$Offset_{X}=0.5\times \left(\mathrm{Accx}\left(-90{}^{\circ}\right)+\mathrm{Accx}\left(+90{}^{\circ}\right)\right)$$

(2) $$Offset_{Y}=0.5\times \left(\mathrm{Accy}\left(0{}^{\circ}\right)+\mathrm{Accy}\left(180{}^{\circ}\right)\right)$$

(3) $$Gain_{X}=0.5\times \left(\frac{(\mathrm{Accx}\left(-90{}^{\circ}\right)+\mathrm{Accx}\left(+90{}^{\circ}\right)}{2}\right)$$

(4) $$Gain_{Y}=0.5\times \left(\frac{(\mathrm{Accy}\left(0{}^{\circ}\right)+\mathrm{Accy}\left(180{}^{\circ}\right)}{2}\right)$$

The calibrated values were calculated according to Equations (5) and (6).

(5) $$Accx,cal=\frac{Accx-Offset_{X}}{Gain_{X}}$$

(6) $$Accy,cal=\frac{Accy-Offset_{Y}}{Gain_{Y}}$$

The angle values were calculated from the measured acceleration values using Equation (6) [47]. For computations, the four-quadrant inverse tangent atan2 function in MATLAB was used [48].

(7) $$\propto =\tan^{-1}\left(\frac{Accx,cal}{Accy,cal}\right)$$

#### 2.3.2. Fluidic Inclination Sensors

**Figure 6:**
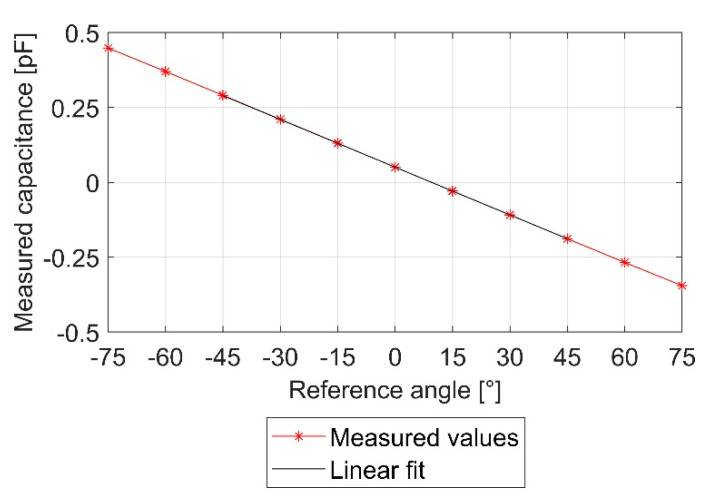
Characteristic curve MID with linear fit.

Figure 6 shows an example for a characteristic curve of a MID sensor with a bias offset at 0°. While several non-idealities cause the offset in most cases, for example tolerances in the circuit design and technology or tolerances due to the manufacturing process of the sensor, adjustments within the circuit design for every single sensor are possible. However, since calibration is necessary in any case, the offset is removed by this method without single sensor adjustments. A linear calibration (Figure 6) was applied to the MID sensors. To calculate gain and offset, three measurement points at −30°, 0°, and 30° were used. To calculate the measured inclination values, Equation (9) was used.

(8) Cap=Gain×α−Offset

(9) $$\alpha =\frac{Cap-Offset}{Gain}$$

where Cap = measured capacitance, and *α* = resulting measured angle.

**Figure 7:**
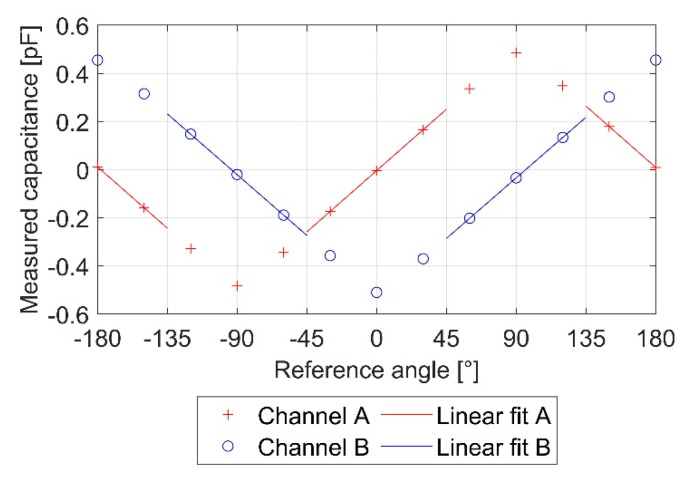
Characteristic curve LP1 with linear fit.

For the sensors PCB1 and PCB2, a partial linear calibration (Figure 7) was applied. Between −180° and −135°, −45° and +45°, and 135° and 180°, the measured capacitance values of Channel A were used. From −135° to −45° and 45° to 135°, Channel B was used.

### 2.4. Allan Deviation

The Allan variance was originally developed for time metrology to measure the frequency and phase stability of clocks [49]. Recently, it became widely used for the evaluation of sensors in general [50]. The Allan variance is, simply expressed, an extended version of the standard deviation for various averaging factors. In this work, the overlapping method of the Allan variance was used for noise analysis and to characterize the stability of the sensors. The overlapping Allan variance was estimated as [51]:

(10) $$\sigma_{y}{}^{2}\left(\tau \right)=\frac{1}{2\left(N-2m\right)\tau^{2}}\overset{N-2m}{\underset{i=1}{\sum }}{\left[x_{i+2m}-2x_{i+m}+x_{i}\right]}^{2}$$

with

(11) $$\tau =m\times \tau_{0}$$

where N = number of samples, m = averaging factor, *τ* = averaging time (seconds), $\tau_{0}$ = measurement interval (seconds), $\sigma_{y}{}^{2}$ = Allan variance, and $x_{\left(t\right)}$ = measured data (°) [51].

The Allan deviation (ADEV) is the square root of the variance [51].

(12) $$\mathrm{ADEV}=\sigma_{y}\left(\tau \right)=\sqrt{\sigma_{y}{}^{2}\left(\tau \right)}$$

**Figure 8:**
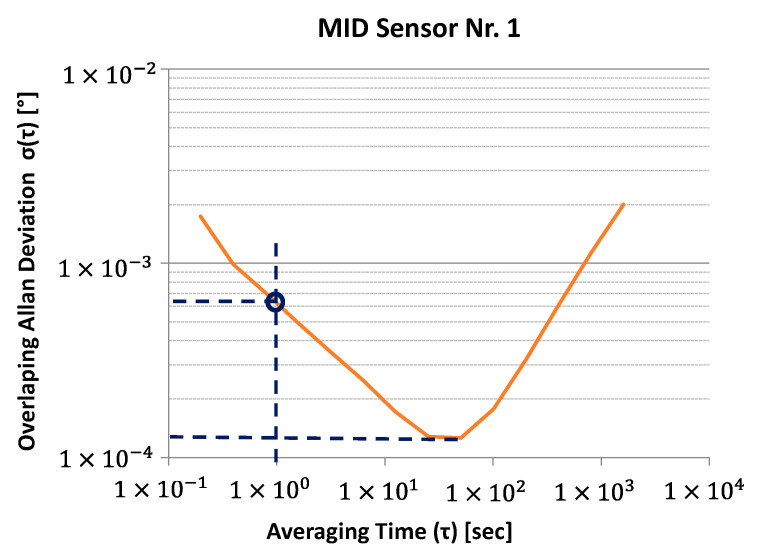
Allan deviation plot with random walk and bias stability.

The result is usually plotted in the log *σ* log *τ* plot and called the Allan deviation plot (Figure 8), where the different types of noise can be identified by their slopes. To obtain the white noise, or random walk, a line is fitted to the curve with the slope of −0.5, and the value is read at the intersection with τ=1. To identify the bias stability, a line with the slope of 0 is fitted to the minimum of the curve [52].

The computations in this work were performed with the freely available program Stable32 [53] from the Institute of Electrical and Electronic Engineers (IEEE) Ultrasonics, Ferroelectrics and Frequency Control Society, and according to the standard IEEE 1139-2008 [49]. The measurements were conducted at a fixed angle of 0° for 2 h. To exclude any influence of the temperature on the measurement results, the measurements took part in the climatic test chamber shown in Figure 5 at constant conditions of 20 °C and 50% relative humidity (RH). A pre-storage was conducted to acclimatize the sensors and for settling of the temperature in the climatic test chamber.

### 2.5. Temperature Stability Offset

**Figure 9:**
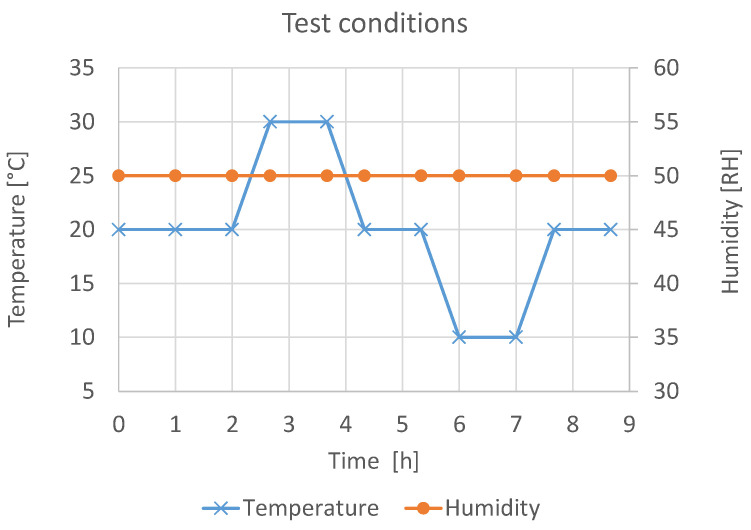
Test conditions for the measurement of temperature stability.

The test setup shown in Figure 5 was used for the measurement of the temperature stability. The temperature during the test was changed between 10 °C and 30 °C, and the relative humidity of 50% was constant, as shown in Figure 9. A pre-storage of at least one hour was conducted to acclimatize the sensors and for settling of the test conditions in the climatic test chamber.

**Figure 10:**
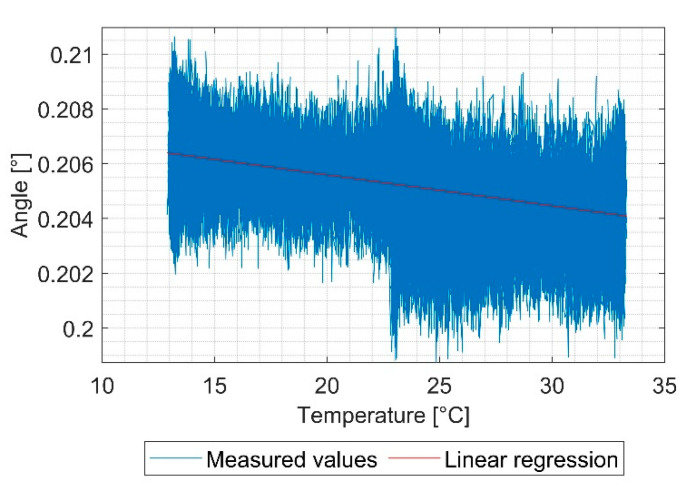
Temperature stability with linear regression with the method of least squares.

During the test, the measured values of the sensors were recorded and afterwards plotted in an angle over temperature diagram, as shown in Figure 10. The slope of the linear fit with the method of least squares was used to obtain the temperature stability.

### 2.6. Characteristic Curve

**Figure 11:**
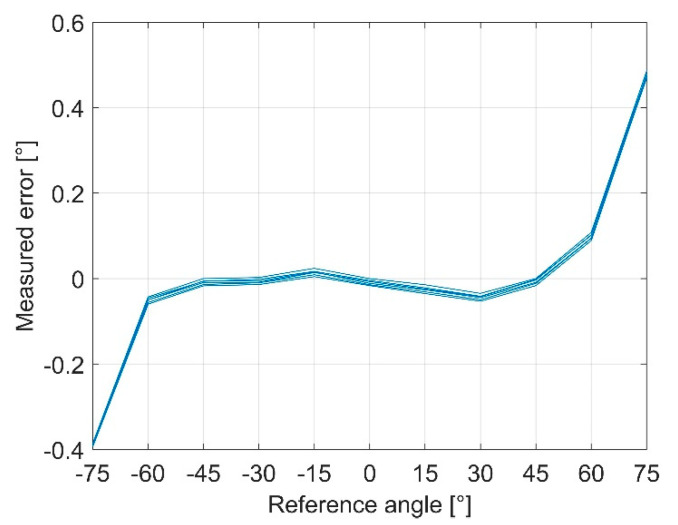
Error curve for the MID sensor measured on the test bench.

The characteristic curves were evaluated according to the international standard IEC61298-2 [54]. The test bench, which is described in Section 2.1, was used to record the data. One test cycle includes three measurement cycles from −180° to +180° and back to 180° with 11 test points each. For the MID sensor, the linear measurement range is ±60°. Calibration and calculation of the measured values are described in Section 2.2. The measurement error is the difference between the reference angle given by the test bench and the angle measured by the sensor (Figure 11).

The non-repeatability and the hysteresis were determined from the error curve and expressed in percent of the full-scale output (% FSO).

The non-repeatability is the maximum difference between the measured values approaching from the same direction at the same measurement point. In contrast, the hysteresis is calculated when approaching the measurement point from a different direction [54].

### 2.7. T-Test

The two-sample *t*-test is a statistical hypothesis test to compare two sets of data with each other. In this work, the independent variant of the two-sample *t*-test with unequal variances and unequal sample sizes was used to compare the results of the measurements of the Allan deviation, the temperature stability, and the characteristic curves of the different sensors. For the calculation, a normal distribution of the measured values was assumed. The results of the *t*-test are the so-called *p*-values, which were calculated with a statistics program. Values of *p* < 0.05 indicate a significant difference between the samples within an approximately 2σ confidence interval [55].

## 3. Results

### 3.1. Fluidic Capacitive Sensors

**Figure 12:**
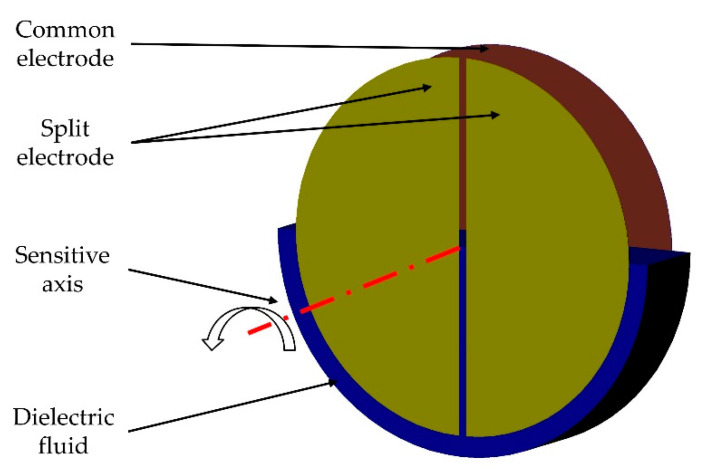
Functional principle with two electrodes in one cavity.

The sensor in this work is based on the principle that surfaces of fluids align horizontally due to gravity. A non-conductive dielectric fluid is partially filled in a circular cylindrical cavity. On the circular ends, one circular and two semicircular electrodes are positioned opposite to each other (Figure 12). The electrodes form a differential capacitor, partially filled with the dielectric fluid. If the inclination changes, the proportion of the surface covered by the fluid changes as well. With a differential capacitive measurement, this change can be detected. The result is a proportional correlation between the angle of inclination and the capacitance.

**Figure 13:**
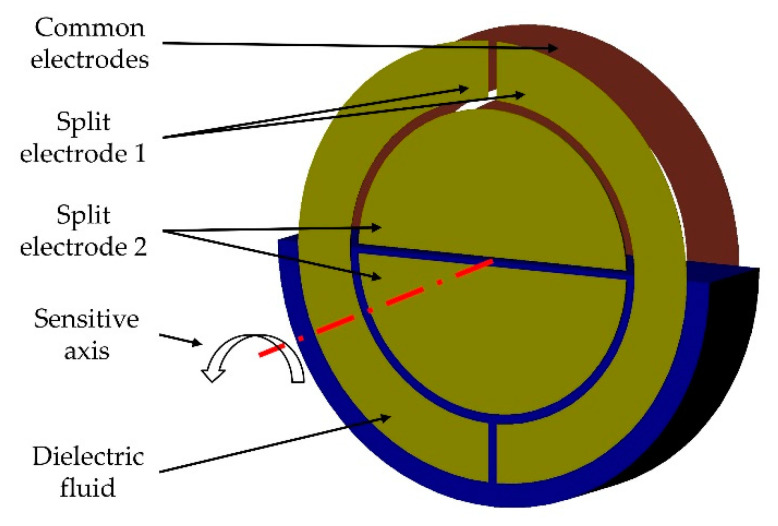
Functional principle with four electrodes in one cavity.

The configuration with two electrodes is limited to a measurement range < ±90°. To overcome that issue, two sensor elements, which are rotated 90° against each other, can be used. In this work, this version is called PCB2. Another possibility is to integrate four electrodes in one cavity (PCB1), as shown in Figure 13.

**Figure 14:**
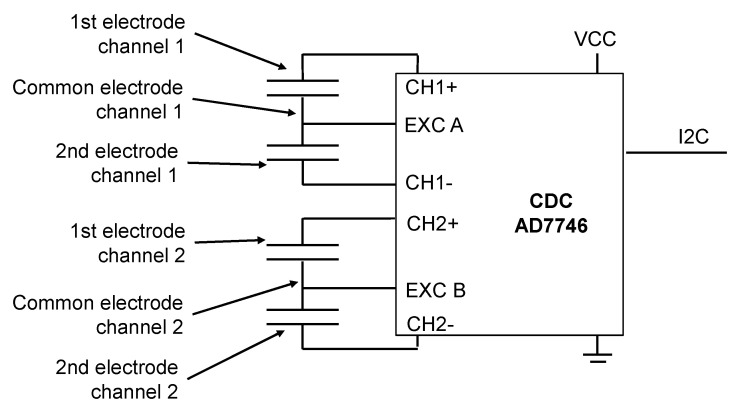
Electrical diagram of the sensors PCB1 and PCB2. The MID sensor only uses channel 1.

The differential capacitive measurement principle is shown in Figure 14. A capacitance to digital converter (CDC) Analog Devices AD7746 is used to measure the capacity. Some technical data of the CDC are given in Table 2. The measurement values are transferred from the CDC via I2C to a microcontroller.

The sensor principle was implemented with two technological approaches: as a molded interconnect device (MID) and using printed circuit boards (PCBs) as the sensor element. The three sensor versions are compared in Table 3.

#### 3.1.1. MID Sensor

The MIDs used in this work are liquid crystal polymer (LCP) injection molded substrates with subtractive structured metallization, i.e., structured by laser ablation. As the MID sensor has one cavity with one pair of electrodes, the measurement range is limited to about ±70°.

**Figure 15:**
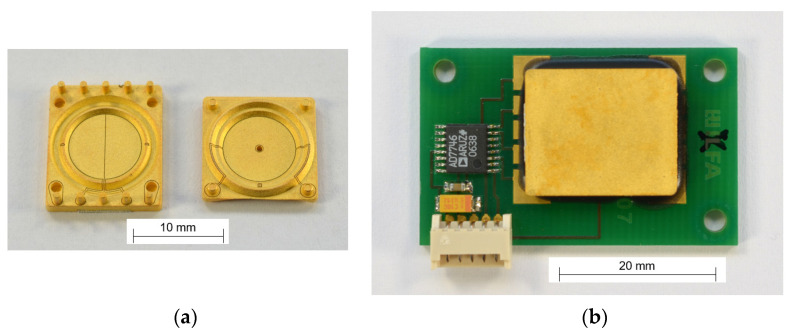
MID version of the fluidic capacitive inclination sensor: (**a**) Components of the sensor element; (**b**) Sensor element with electronics on PCB.

The production steps for the MID sensor (Figure 15) are:

(1) Injection molding of the substrate;
(2) Full-surface chemical metallization of the substrate with copper;
(3) Subtractive laser structuring of the metallization;
(4) Reinforcing of the metallization with nickel and gold for diffusion and corrosion protection;
(5) Adhesive bonding of the two halves of the sensor element to form the cavity;
(6) Filling the cavity halfway with the dielectric fluid;
(7) Closing of the filling hole by soldering;
(8) Placing and soldering of the electronic components on the PCB;
(9) Electrical connection of the sensor element on the PCB with isotropic conductive adhesive (ICA);
(10) Application of an epoxy underfiller for mechanical stability.

#### 3.1.2. PCB Sensor

**Figure 16:**
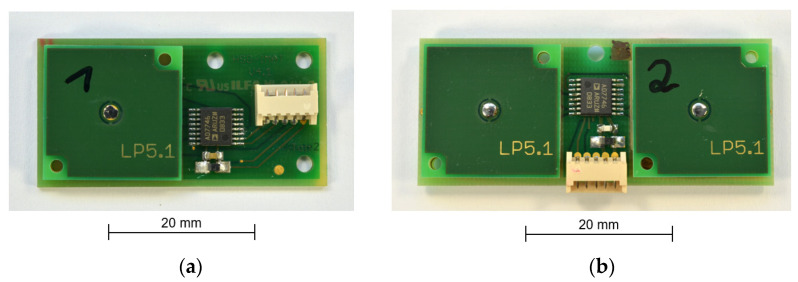
PCB variant of the sensor: (**a**) PCB1; (**b**) PCB2.

The two versions of the PCB sensor offer a measurement range of 360° and are shown in Figure 16. PCB1 uses two pairs of electrodes in one cavity, as described in Figure 13. PCB2 uses two sensor elements with one pair of electrodes (Figure 12) each, which are rotated 90° against each other.

**Figure 17:**
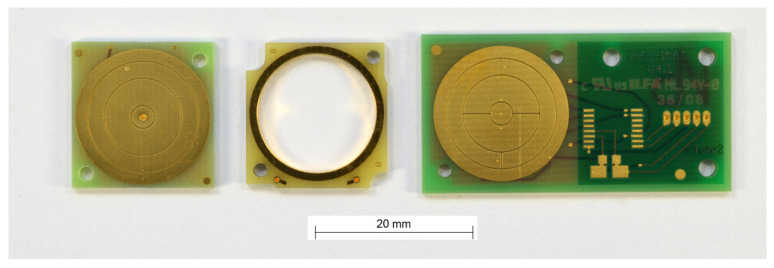
Components of the PCB sensor; top, middle, and bottom PCB.

The production steps for both PCB sensor variants are:

(1) Dispensing of solder paste on the bottom and middle PCBs (Figure 17);
(2) Stacking of the middle and the top PCB on the bottom PCB;
(3) Placement of electronic components on the PCB;
(4) Reflow soldering;
(5) Filling the cavity halfway with the dielectric fluid;
(6) Closing of the filling hole by soldering;

### 3.2. Allan Deviation

**Figure 18:**
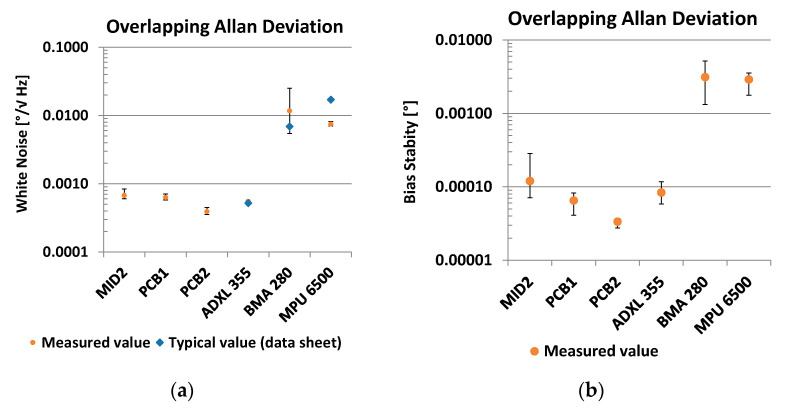
Allan deviation: (**a**) White noise; (**b**) Bias stability.

Two characteristic values are obtained from the measurement of the Allan deviation: white noise (Figure 18a) and bias stability (Figure 18b). The complete Allan plots can be found in the Supplementary Materials.

The white noise of the PCB2 sensors (mean = 0.00039; SD = 3.9 × 10^−5^) is significantly less (t(10) = −9.5; *p* = 2.5 × 10^−6^) than the white noise of the ADXL355 sensors (mean = 0.00054; SD = 2.1 × 10^−5^). The measurement results for the ADXL355 match with the specification in the data sheet of 9 µg/√Hz = 0.00052°/√Hz (typical value) [34]. Compared to the other fluidic sensors PCB1 (mean = 0.0006; SD = 4.5 × 10^−5^; t(11) = −11.0; *p* = 2.9 × 10^−7^) and MID (mean = 0.00067; SD = 7.9 × 10^−5^; t(8) = −8 × 6; *p* = 2.5 × 10^−5^), the PCB2 variant performs best as well. The measured values for the sensors BMA280 and MPU 280 are one order of magnitude higher.

Regarding bias stability, a similar result is observed. The PCB2 sensors (mean = 3.4 × 10^−5^; SD = 3.7 × 10^−6^) perform significantly better compared to the ADXL355 sensors (mean = 8.3 × 10^−5^; SD = 2.1 × 10^−5^; t(4) = −5.2; *p* = 0.007) and compared to the PCB1 sensors (mean = 6.5 × 10^−5^; SD = 1.4 × 10^−5^; t(7) = −5.5; *p* = 0.0009). An evaluation in tabular form can be found in the Supplementary Materials.

### 3.3. Temperature Stability

**Figure 19:**
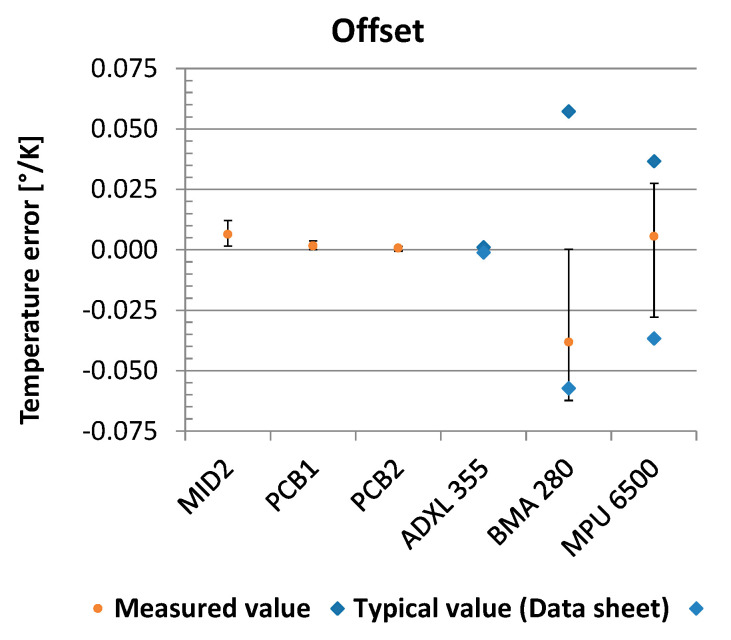
Temperature error sensor offset.

The measured temperature errors (Figure 19) for the different sensors span several orders of magnitude. Among the fluidic sensors, the PCB2 variant performs with an average offset temperature error of 0.0007°/K (SD = 0.0006) best. The sensor ADXL355 is slightly (mean = −0.0002; SD = 0.0003) but significantly (t(9) = 3.2; *p* = 0.01) better. For the sensors BMA280 (mean = −0.038; SD = 0.026) and MPU6500 (mean = 0.006; SD = 0.021), the measured values are way higher than for all the others; they scatter strongly, and they are within the data sheet specifications [35,36]. An evaluation in tabular form can be found in the Supplementary Materials.

### 3.4. Characteristic Curve

**Figure 20:**
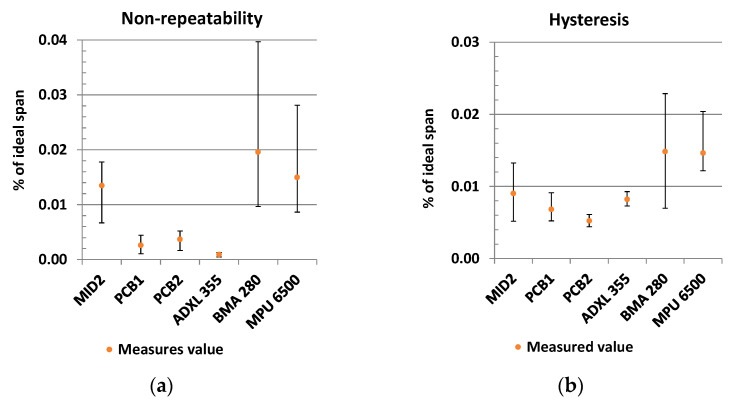
Characteristic curve: (**a**) Non-repeatability; (**b**) Hysteresis.

The characteristic values hysteresis (Figure 20a) and non-repeatability (Figure 20b) are obtained from the characteristic curve.

Regarding non-repeatability, the MEMS sensor ADXL355 (mean = 0.0009; SD = 0.0004) performs significantly better than the PCB1 (mean = 0.0026; SD = 0.0013; t(7)= 3.4; *p* = 0.01) and the PCB2 sensors (mean = 0.004; SD = 0.0013; t(7) = 5.2; *p* = 0.001).The MID variant is ranked clearly behind them.

The measurement results of the hysteresis rank the other way around for the fluidic sensors and the ADXL355 MEMS. The PCB2 sensors (mean = 0.0052; SD = 0.0006) perform significantly better than the PCB1 (mean = 0.0068; SD = 0.0012; t(9) = −3.1; *p* = 0.01) and the ADXL355 (mean = 0.0082; SD = 0.0008; t(7) = −6.8; *p* = 0.0003) sensors.

The measured values for the MEMS sensors Bosch BMA280 and MPU6500 show, for both, non-repeatability and hysteresis, on average higher values, and they scatter strongly. An evaluation in tabular form can be found in the Supplementary Materials.

## 4. Discussion

**Figure 21:**
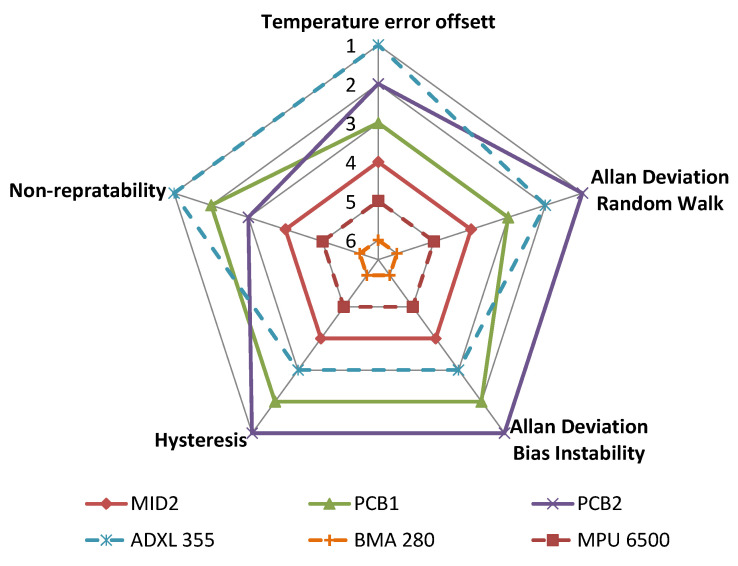
Comparison.

Figure 21 summarizes the results of the benchmark. The two MEMS sensors BMA280 and MPU6500 rank last in each studied category. They are followed by the MID variant of the fluidic sensor. The fluidic sensors PCB1 and PCB2 and the MEMS sensor ADXL355 perform best. Comparing these three sensors, each has individual qualities. The silicon MEMS sensor ADXL355 offers the lowest offset temperature error and non-repeatability. Regarding random walk, white noise, and hysteresis, the PCB2 sensor ranks best.

Hysteresis is one of the most important parameters for estimating the achievable overall accuracy, since it cannot be improved by any calibration. Hysteresis is well below 0.01% FSO = 0.036° for both fluidic PCB sensors. This qualifies them to be considered for many applications.

Regarding costs, the MID sensor should result in the highest costs of the three variants of the fluidic capacitive sensors. The production of the MID elements requires injection molding, laser structuring, and metallization. Laser structuring and metallization are not common in SMEs. Injection molding requires high tooling costs compared to the purchase of printed circuit boards. As a result, small series become uneconomical, and adaptation to other sensor sizes is costly.

Since only PCBs are required for the sensor cell, the PCB sensors can be manufactured more economically than the MID sensors. The cost difference between the two PCB variants is small. The second sensor cell for the PCB2 sensor requires only more PCBs and the process cost for filling the second cavity. However, the size difference could be relevant for some applications.

The fluidic capacitive sensors cannot compete with the costs of the BMA280 and the MPU6500 sensors. However, the benchmark has demonstrated that the performance of these sensors does not match the performance of the fluidic sensors. Thus, different applications with higher requirements can be addressed. Another advantage of MEMS is their small size, which is particularly relevant in mobile devices or other consumer electronics. In these applications, however, long-term stability and temperature stability of the sensor signal are of secondary importance.

The main advantages of the fluidic capacitive inclination sensor are the lowest bias stability, white noise, and hysteresis, compared to the other sensors in the benchmark. These properties are very important in some applications. Examples can be found in the field of construction, land surveying, renewable energies, and many more. Monitoring of horizontal alignment construction vehicles such as excavators, cranes, and lifting platforms are examples in the construction industry. In surveying technology, the fluidic sensor can enhance the function of laser rangefinders by triangulating heights or projecting lengths. In the field of renewable energies, possible applications are the monitoring of wind turbines or the alignment of solar cells in solar trackers and concentrated solar thermal collectors to the position of the sun.

**Proof** **of** **Theorem 1.** Due to the better performance compared to the BMM MEMS sensors BMA280 and MPU6500 and the overall comparable performance to the ADXL355 sensor, the theorem of a favorable market position for the fluidic capacitive inclination sensors can be confirmed. □

The systematic temperature behavior is a main advantage of the fluidic principle compared to the statistical temperature behavior of MEMS accelerometers. Further research could include more sophisticated calibration methods for the fluidic capacitive inclination sensor. The temperature effect in the sensor cell is systematic due to the thermal expansion of the dielectric fluid and the resulting change in permittivity. Thus, it can be compensated by a suitable temperature calibration that calibration can be determined based on some samples, which are characterized within the operating temperature range. The result can be generalized afterwards for all other sensors.

Another possibility for further research could be a sensor fusion of the fluidic inclination sensor with an MEMS gyroscope. The fluidic sensor offers good long-term stability. Due to the viscosity of the dielectric fluid, dynamic applications are limited. MEMS gyroscopes offer a temporal highly resolved measurement of the angular velocity. The fusion of both signals should result in a long-term stable and simultaneously highly dynamic inclination sensor.

The CDC AD7746 from Analog Devices was used in this work for capacitance measurement. There are other possibilities for capacitance measurement on the market, e.g., SioSense PCap01, Texas Instruments FDC1004, and others. Further research could compare sensors with the same fluidic capacitive sensor cell (e.g., PCB1 or PCB2) and different CDCs.

## 5. Conclusions

This investigation compares a novel fluidic capacitive inclination sensor with commercially available sensors. Different variants of the fluidic sensor and different integrated circuit packaging technologies are presented. Within the commercially available sensors, three silicon MEMS acceleration sensors in different price ranges, representing the targeted market segment, were chosen.

Among the two production technologies for the fluidic sensor cell, the stacking of PCBs is more favorable than the MID technology, as only commonly available SMT is needed. This results in a shorter process chain. Additionally, the performance is better, and the measurement range is larger.

The key findings of the benchmark are: the MEMS sensor ADXL355 has significantly better characteristic values compared to the MEMS sensors BMA280 and MPU6500. The fluidic sensors outperform the cheaper MEMS in every characterized category. Compared to the ADXL355 sensors especially, the PCB variant of the fluidic sensor performs equally well or even better in some categories.

Overall, it can thus be concluded that the sensor has at least a niche position, possibly more, in the market.

## Figures and Tables

**Table 1:** Commercial silicon MEMS acceleration sensors.

Sensor	Analog Devices ADXL355 [34]	Bosch BMA280 [35]	TDK InvenSense MPU6500 [36]	Safran Colibrys SI1003 [37]
Technology	SMM	SMM	SMM	BMM
No. of axes	3	3	3 ^1^	1
Measurement range	±2 g	±2 g	±2 g	±3 g
Noise	22.5 µg/√Hz	120 µg/√Hz	300 µg/√Hz	0.7 µg/√Hz
Bias temperature coefficient	±0.15 mg/K	±1.0 mg/K	±0.6 mg/K	±0.3 mg/K
Output	Digital	Digital	Digital	Analog (voltage)
Package	Ceramic LCC14	Polymer LGA	Polymer QFN	Ceramic LCC20
Package size	6 × 5.6 × 2.2 mm^3^	2 × 2 × 0.95 mm^3^	3 × 3 × 0.9 mm^3^	8.9 × 8.9 × 3.2 mm^3^
Evaluation board size ^2^	20 × 20 × 5 mm^3^	43 × 20 × 3 mm^3^	26 × 15 × 3 mm^3^	
Cost: Purchase quantity 500 items	35.95 €/Item [38]	0.99 €/Item [39]	3.63 €/Item [40]	279.74 €/Item [41]

^1^ The sensor is an integrated three-axis accelerometer and a three-axis gyroscope. In this work, only the accelerometer is used; ^2^ Without connector.

**Table 2:** AD7746 specifications; Data extracted from [56].

Quantity	Value
Measurement range	±4 pF
Measurement mode	Floating
Number of channels	2 (multiplexed)
Update rate	10 … 90 Hz
Output noise, rms	2 aF/√Hz
Interface	I2C
Temperature range	−40 … +125 °C
Temperature sensor	Integrated or external RTD, thermistor, or diode ^1^

^1^ For temperature measurement, the on-chip temperature sensor was used.

**Table 3:** Fluidic sensors.

Sensor	MID	PCB 1	PCB 2
Technology	MID	Stacked PCB	Stacked PCB
No. of sensor elements	1	1	2
Measurement range	±60°	360°	360°
Size sensor element	13 × 16 × 3 mm^3^	18 × 18 × 5 mm^3^	18 × 18 × 5 mm^3^
Size sensor with PCB ^1^	33 × 21 × 5 mm^3^	39 × 20 × 5 mm^3^	48 × 20 × 5 mm^3^
CDC	Analog Devices AD7746, 24 bit		
Data rate	5.4 Hz	5.1 Hz	5.1 Hz
Estimated costs ^2^	~30 €	~19 €	~20 €

^1^ Without connector; ^2^ Estimation can be found in the Supplementary Materials.

## Data Availability

All data used are shown in the text and the Supplementary Materials. Raw data are available on request.

## Supplementary Materials

The following are available online at https://www.mdpi.com/article/10.3390/s21238030/s1, Figure S1. Table S1: Cost estimation for the MID, PCB1 and PCB2 sensors, Figure S2. Allan plot Analog Devices ADXL355, Figure S3. Allan plot Bosch BMA280, Figure S4. Allan plot MID, Figure S5. Allan plot LP1, Figure S6. Allan plot LP2, Figure S7. Allan plot TDK InvenSense MPU6500, Table S1: White noise, Table S2: Bias stability, Table S3: Temperature stability offset, Table S4: Non-repeatability, Table S5: Hysteresis.
