# Supplementary material for: Characterization and Benchmark of a Novel Capacitive and Fluidic Inclination Sensor
Source: Sensors (Basel). 2021 Dec 1;21(23):8030. doi: 10.3390/s21238030 (PMC8659599; doi:10.3390/s21238030)
Supplement: Supplementary file 1 [file sensors-21-08030-s001.zip › sensors-1451866-supplementary.pdf]

sensors

MDPI

Supplementary Materials

Characterization and Benchmark of a Novel Capacitive and Fluidic Inclination Sensor

Table S1: Cost estimation for the MID, PCB1 and PCB2 sensors

	**MID**	**PCB1**	**PCB2**
**CDC**	8,23 €	8,23 €	8,23 €
**Passive components**	0,08 €	0,08 €	0,08 €
**PCBs**	0,13 €	0,95 €	1,49 €
**MIDs**	≈ 5,00 €	x	x
**Sum material costs,**	≈ 15,00 €	9,35 €	9,89 €
**Overall sensor costs including packaging and test (lot size: 2,500 units)**	≈ 30,00 €	≈ 19,00 €	≈ 20,00 €

The total costs were estimated using the formula of thumb that material costs are roughly 50% of the total costs and manufacturing, packaging, and test are the other 50%.

Material costs were estimated by researching prices from the major distributors Arrow, Digikey, Farnell, Mouser, and RS Components, and PCB costs were estimated using online configurators from Würth, Lite-On, and PCBWay. Table S1 shows the most favorable offers in each case.

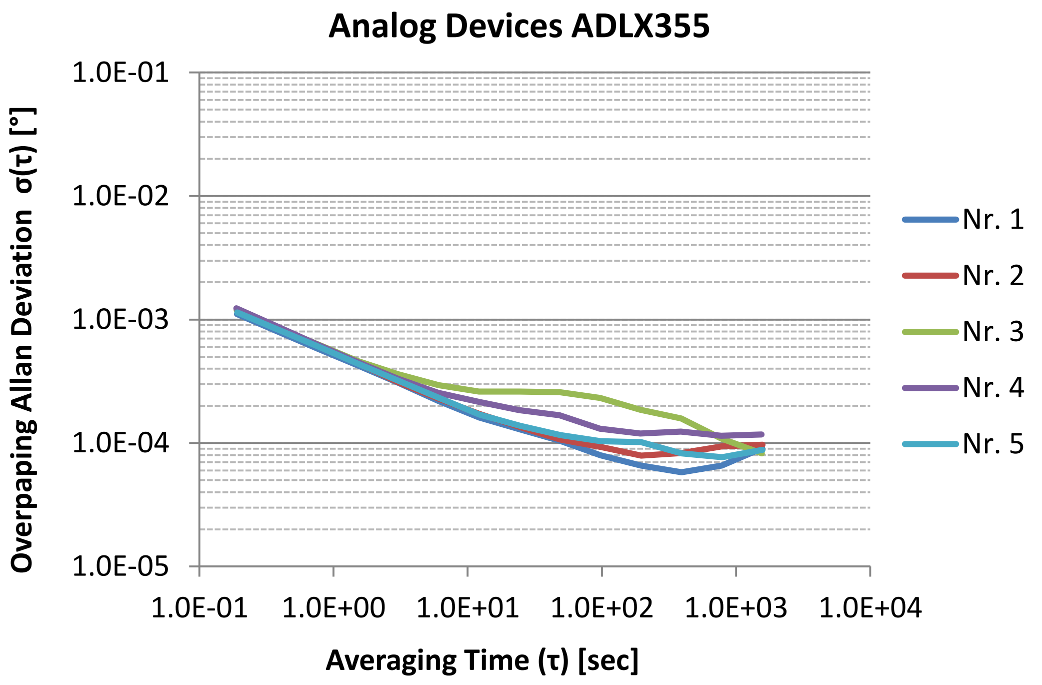

Figure S1. Allan plot Analog Devices ADXL355

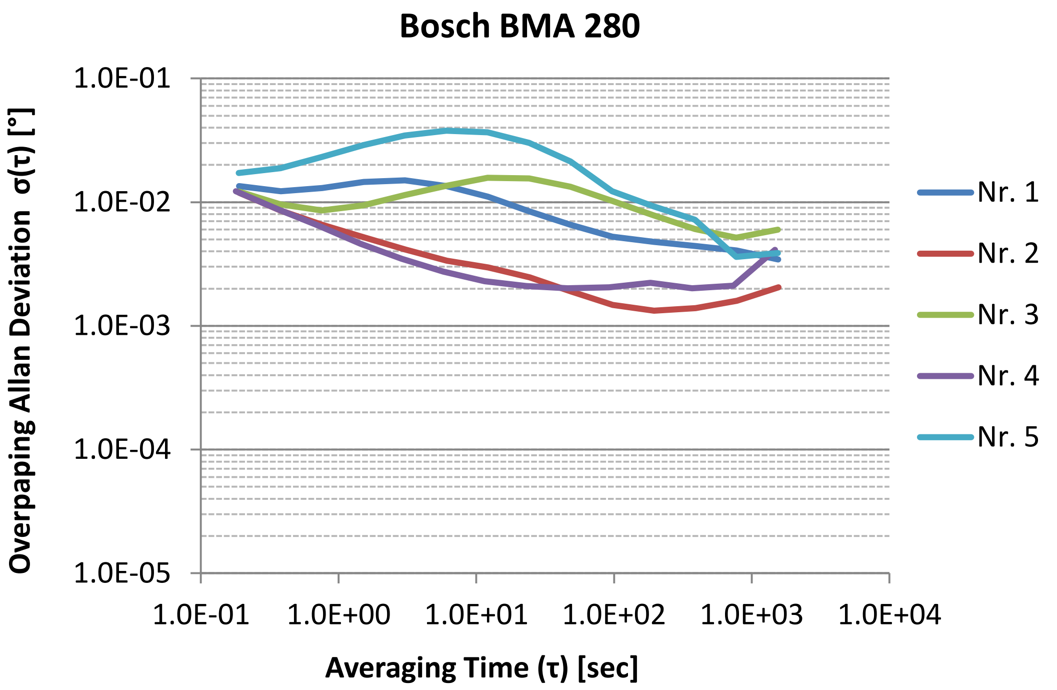

Figure S2. Allan plot Bosch BMA280

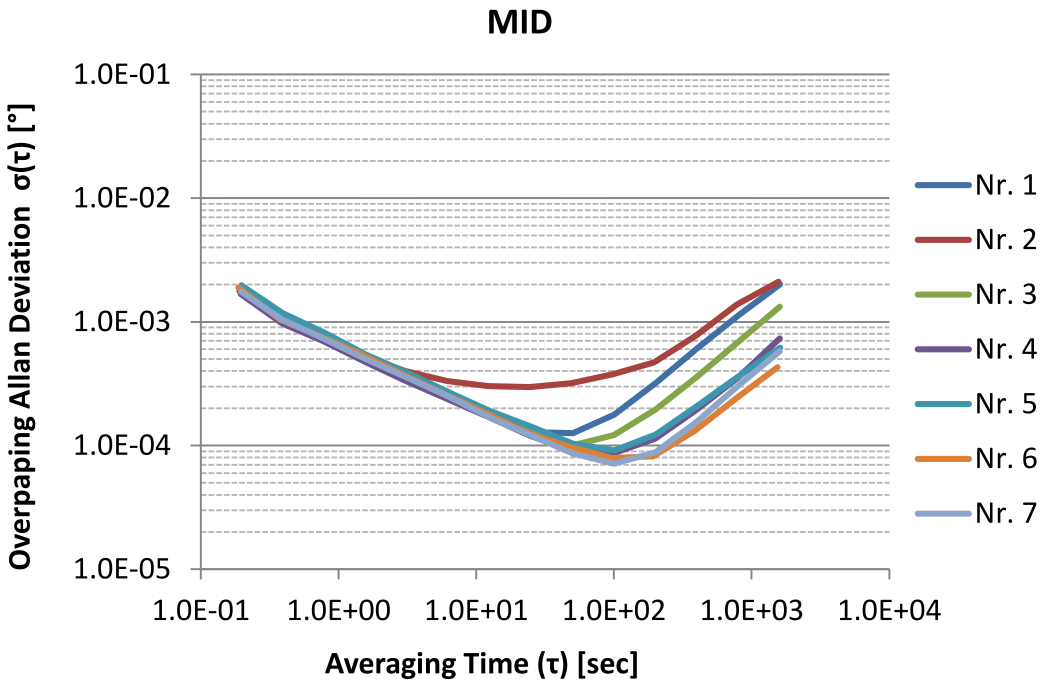

Figure S3. Allan plot MID

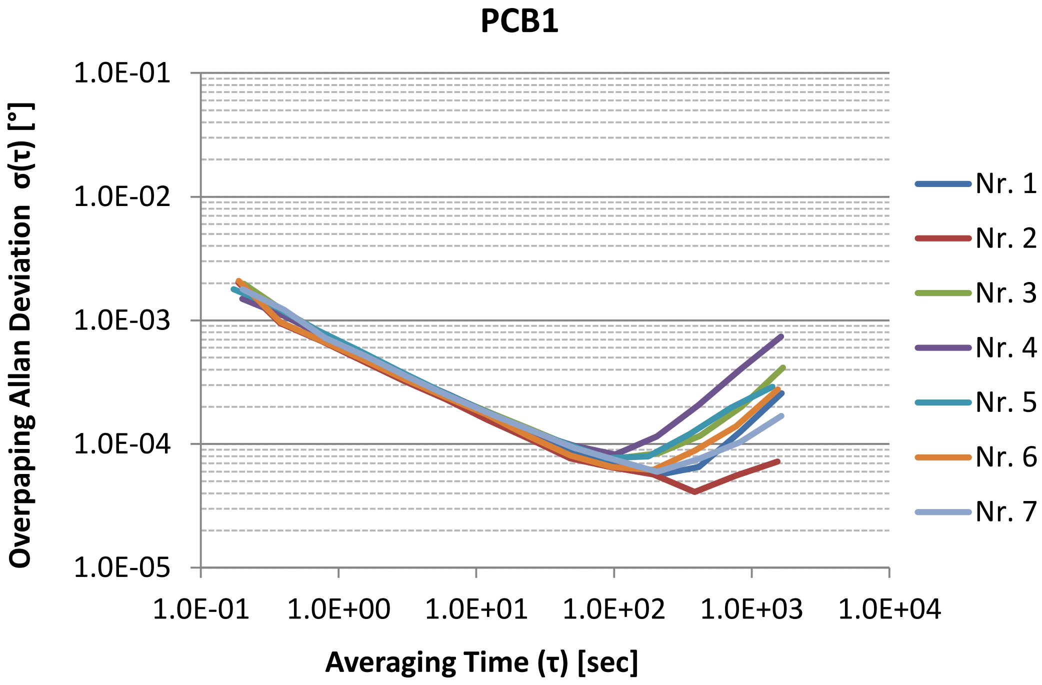

Figure S4. Allan plot LP1

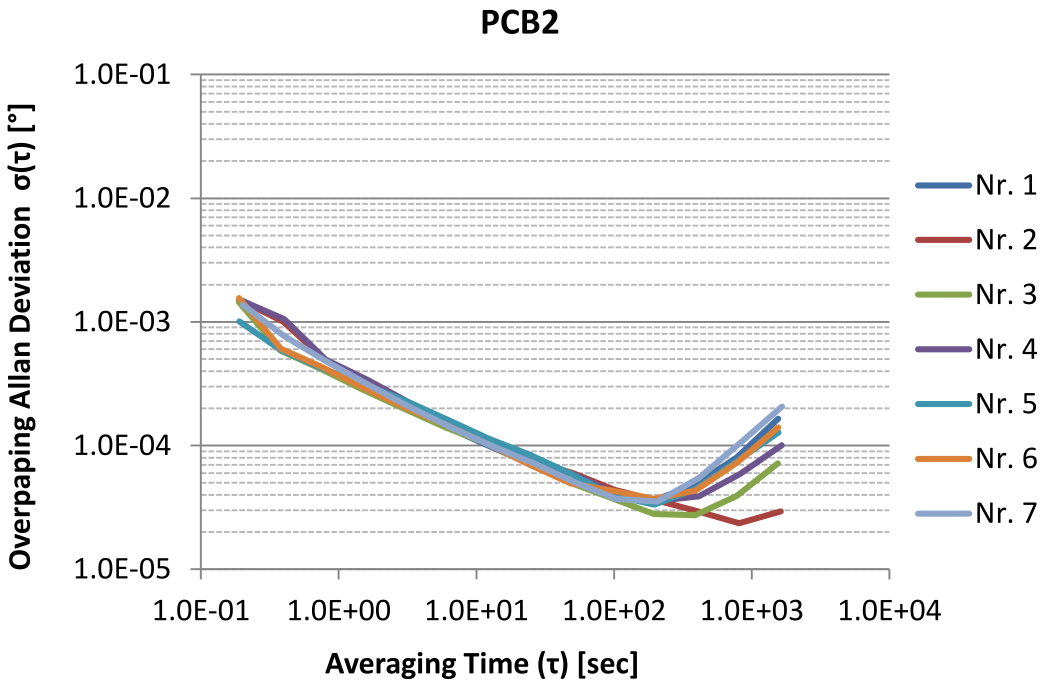

Figure S5. Allan plot LP2

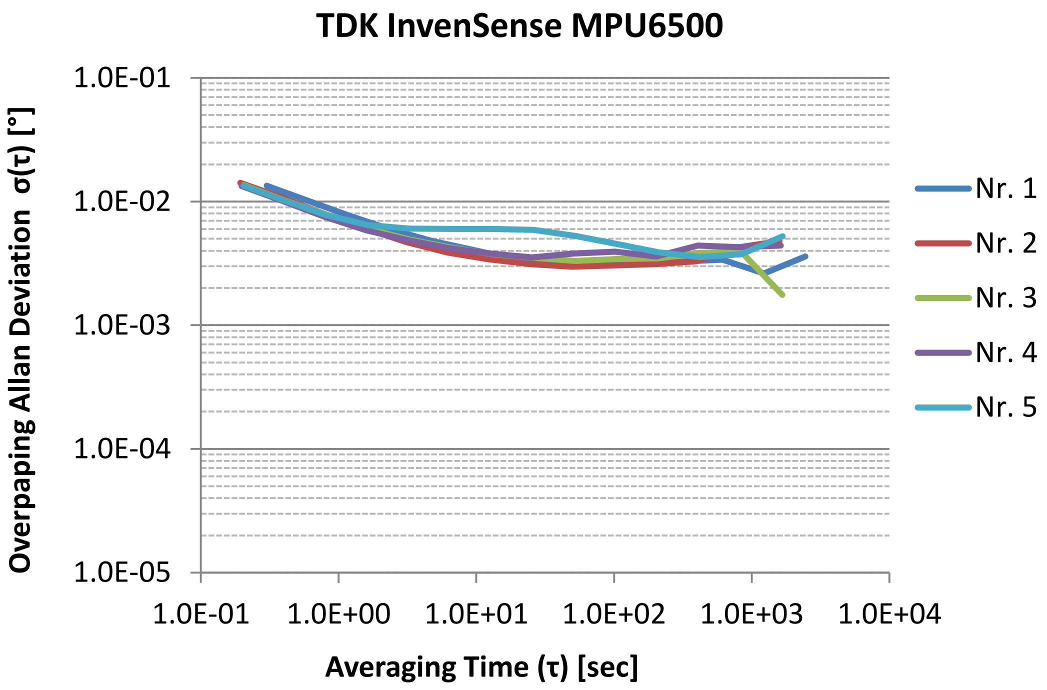

Figure S6. Allan plot TDK InvenSense MPU6500

Table S2: White noise

Sensor		MID2	PCB1	PCB2	ADXL 355	BMA 280	MPU 6500
					White Noise		
Messwert Sample 1	[°/√Hz]	0.00063	0.00060	0.00036	0.00052	0.01300	0.00820
Messwert Sample 2	[°/√Hz]	0.00084	0.00058	0.00041	0.00054	0.00600	0.00727
Messwert Sample 3	[°/√Hz]	0.00065	0.00065	0.00035	0.00057	0.00900	0.00735
Messwert Sample 4	[°/√Hz]	0.00060	0.00064	0.00042	0.00057	0.00547	0.00699
Messwert Sample 5	[°/√Hz]	0.00071	0.00071	0.00038	0.00054	0.02500	0.00754
Messwert Sample 6	[°/√Hz]	0.00065	0.00059	0.00037			
Messwert Sample 7	[°/√Hz]	0.00064	0.00064	0.00045			
Mittelwert	[°/√Hz]	0.00067	0.00063	0.00039	0.00055	0.01169	0.00747
Standardabweichung	[°/√Hz]	0.000079	0.000045	0.000036	0.000021	0.008018	0.000454
Typical value (data sheet)	[°/√Hz]				0.00052	0.00688	0.01700
Typical value (data sheet)	[µg/√Hz]				9	120	220

Table S3: Bias stability

Sensor		MID2	PCB1	PCB2	ADXL 355	BMA 280	MPU 6500
					**Bias Stability [°]**		
Measured value sample 1	[°]	0.00013	0.00006	0.00004	0.00006	0.00344	0.00263
Measured value sample 2	[°]	0.00029	0.00004	0.00003	0.00008	0.00132	0.00298
Measured value sample 3	[°]	0.00010	0.00008	0.00003	0.00008	0.00517	0.00176
Measured value sample 4	[°]	0.00009	0.00008	0.00004	0.00012	0.00201	0.00354
Measured value sample 5	[°]	0.00009	0.00008	0.00003	0.00008	0.00361	0.00355
Measured value sample 6	[°]	0.00008	0.00006	0.00004			
Measured value sample 7	[°]	0.00007	0.00006	0.00004			
Mean value	[°]	0.00012	0.00007	0.00003	0.00008	0.00311	0.00289
Standard Deviation	[°]	0.000075	0.000015	0.0000038	0.000021	0.001501	0.000743
Typical value (data sheet)	[°]				0.00011		
Typical value (data sheet)	[mg]				2		

Table S4: Temperature stability offset

Sensor		MID2	PCB1	PCB2	ADXL 355	BMA 280	MPU 6500
					**Temperature Stability Offset**		
Measured value sample 1	[°/K]	0.0056	0.0009	0.0010	-0.0001	-0.062	0.002
Measured value sample 2	[°/K]	0.0095	0.0000	-0.0005	0.0003	-0.062	0.019
Measured value sample 3	[°/K]	0.0091	0.0035	0.0013	-0.0002	-0.035	0.008
Measured value sample 4	[°/K]	0.0121	0.0000	0.0012	-0.0003	0.000	-0.028
Measured value sample 5	[°/K]	0.0016	0.0004	0.0006	-0.0005	-0.031	0.027
Measured value sample 6	[°/K]	0.0038	0.0033	0.0010			
Measured value sample 7	[°/K]	0.0035	0.0036	0.0004			
Mean value	[°/K]	0.0065	0.0017	0.0007	-0.00015	-0.038	0.006
Standard Deviation	[°/K]	0.0038	0.0017	0.0006	0.0003	0.026	0.021
Typical value (data sheet)	[°/K]				± 0,001	± 0,057	± 0,037
Typical value (data sheet)	[mg/K]				± 0,02	±1	±0,64

Table S5: Non-repeatability

Sensor		Non-repeatability MID2	PCB1	PCB2	ADXL 355	BMA 280	MPU 6500
Messwert Sample 1	[% FS]	0.0158	0.0011	0.0049	0.0005	0.0156	0.0088
Messwert Sample 2	[% FS]	0.0067	0.0044	0.0017	0.0013	0.0097	0.0159
Messwert Sample 3	[% FS]	0.0111	0.0026	0.0044	0.0010	0.0225	0.0086
Messwert Sample 4	[% FS]	0.0178	0.0038	0.0036	0.0006	0.0103	0.0134
Messwert Sample 5	[% FS]	0.0121	0.0017	0.0022	0.0013	0.0397	0.0281
Messwert Sample 6	[% FS]	0.0152	0.0015	0.0052			
Messwert Sample 7	[% FS]	0.0156	0.0032	0.0037			
Mittelwert	[% FS]	0.0135	0.0026	0.0037	0.00092	0.0196	0.0150
Standardabweichung	[% FS]	0.0038	0.0013	0.0013	0.00037	0.0124	0.0080

Table S6: Hysteresis

Sensor		Hysteresis MID2	PCB1	PCB2	ADXL 355	BMA 280	MPU 6500
Messwert Sample 1	[% FS]	0.013	0.006	0.006	0.008	0.017	0.014
Messwert Sample 2	[% FS]	0.005	0.005	0.006	0.009	0.007	0.013
Messwert Sample 3	[% FS]	0.008	0.007	0.006	0.008	0.017	0.014
Messwert Sample 4	[% FS]	0.012	0.007	0.005	0.007	0.010	0.020
Messwert Sample 5	[% FS]	0.010	0.006	0.004	0.009	0.023	0.012
Messwert Sample 6	[% FS]	0.007	0.009	0.005			
Messwert Sample 7	[% FS]	0.008	0.007	0.005			
Mittelwert	[% FS]	0.009	0.007	0.005	0.008	0.015	0.015
Standardabweichung	[% FS]	0.003	0.001	0.001	0.001	0.006	0.003
